# Virtual screening and network pharmacology-based synergistic mechanism identification of multiple components contained in Guanxin V against coronary artery disease

**DOI:** 10.1186/s12906-020-03133-w

**Published:** 2020-11-13

**Authors:** Bo Liang, Xiao-Xiao Zhang, Ning Gu

**Affiliations:** 1 Nanjing University of Chinese Medicine, Nanjing, China; 2 Nanjing Hospital of Chinese Medicine Affiliated to Nanjing University of Chinese Medicine, Nanjing, China

**Keywords:** Guanxin V, Coronary artery disease, Virtual screening, Network pharmacology, HPLC

## Abstract

**Background:**

Guanxin V (GXV), a traditional Chinese medicine (TCM), has been widely used to treat coronary artery disease (CAD) in clinical practice in China. However, research on the active components and underlying mechanisms of GXV in CAD is still scarce.

**Methods:**

A virtual screening and network pharmacological approach was utilized for predicting the pharmacological mechanisms of GXV in CAD. The active compounds of GXV based on various TCM-related databases were selected and then the potential targets of these compounds were identified. Then, after the CAD targets were built through nine databases, a PPI network was constructed based on the matching GXV and CAD potential targets, and the hub targets were screened by MCODE. Moreover, Metascape was applied to GO and KEGG functional enrichment. Finally, HPLC fingerprints of GXV were established.

**Results:**

A total of 119 active components and 121 potential targets shared between CAD and GXV were obtained. The results of functional enrichment indicated that several GO biological processes and KEGG pathways of GXV mostly participated in the therapeutic mechanisms. Furthermore, 7 hub MCODEs of GXV were collected as potential targets, implying the complex effects of GXV-mediated protection against CAD. Six specific chemicals were identified.

**Conclusion:**

GXV could be employed for CAD through molecular mechanisms, involving complex interactions between multiple compounds and targets, as predicted by virtual screening and network pharmacology. Our study provides a new TCM for the treatment of CAD and deepens the understanding of the molecular mechanisms of GXV against CAD.

**Supplementary Information:**

The online version contains supplementary material available at 10.1186/s12906-020-03133-w.

## Background

Even though interrelated changes in demography, environment, lifestyle, and health care, including the rising burden from coronary artery disease (CAD), indicate a transition in cardiovascular diseases epidemiology, cardiovascular diseases are still the leading cause of death worldwide [1, 2], posing a huge threat to global public health [3, 4]. Among cardiovascular death, CAD accounts for a large part [4, 5]. The treatment of CAD is mainly based on lifestyle management, drug treatment, and revascularization [6]. Guanxin V (GXV), an in-hospital preparation traditional Chinese medicine (TCM), has a significant effect on CAD [7–13], and our previous studies showed that GXV reduced serum inflammatory factor release [10, 14–17] through the TLR4/MyD88/NF-κB signaling pathway [16, 18, 19]. Network pharmacology, as a multidisciplinary science, reveals the pathophysiology and therapy strategies of numerous disorders by integrating related sciences [20–22], such as systems biology and pharmacology. Network pharmacology is of great significance for the discovery of effective components and potential targets in TCM and the investigation of its underlying mechanisms, which may help explore the pharmacological properties of herbal medicines [23, 24]. In view of the complex composition of TCM, it has the characteristics of multitarget, multichannel and coordination and synergism [25–28]. With the continuous improvement in the natural science system, the study of in-depth drug disease mechanisms is increasing [29]. However, due to the relatively backward research on the modernization of TCM theory, the integrity of TCM, the complexity of TCM components and the multichannel and multitarget mechanisms of the property, the material basis of the efficacy of TCM is not clear, and the mechanisms are not clear. At present, the mechanisms of GXV in CAD have not been fully elucidated, except for the previously mentioned mechanisms. In the present study, virtual screening and network pharmacology-based synergistic mechanism investigations of multiple components contained in GXV against CAD were systematically conducted.

## Materials and methods

### Compounds in GXV

The chemical compounds contained in the six herbs (*Codonopsis Radix*, *Ophiopogon japonicus*, *Schizandra Chinensis Fructus*, *Rehmannia Radix Praeparata*, *Radix Salviae*, and *Radix Paeoniae Rubra*) that constitute GXV were retrieved from TCMSP [30], TCMID [31], and BATMAN-TCM [32]. In addition, we combined the published literature to supplement the chemical compounds of the drugs contained in GXV.

### Active compounds of GXV

The ADME characteristics of each obtained chemical compound were explored to obtain the bioactive compounds in six herbs of GXV. In our study, oral bioavailability (OB) [30, 33] and drug likeness (DL) [30, 34], two commonly used ADME-related parameters, were assessed for each bioactive chemical compound. Generally, compounds with OB ≥ 30% and DL ≥ 0.18 are considered to have pharmacological activities [35, 36].

### Targets of active compounds in GXV

After obtaining the active compounds in GXV, the potential targets were also investigated from TCMSP [30], TCMID [31], and BATMAN-TCM [32]. Similarly, we combined the published literature to supplement the targets of active compounds in GXV.

### Targets in CAD

The CAD-associated human targets were surveyed using diverse databases, including GeneCards [37], TTD [38], DrugBank [39], DisGeNET [40, 41], OMIM, TCMSP [30], MalaCards [42], NCBI, and CTD [43] with the search species limited to ‘*Homo sapiens*’. Among them, candidate targets with correlation scores ≥ the mean in GeneCards [37] and gene disease correlation scores ≥ the mean in DisGeNET [40, 41] were included in the follow-up analysis. Furthermore, the potential targets were supplemented with relevant literature, and all the results were summarized and deduplicated.

### H-C, H-C-T, and H-C-T-D networks

The herb-compound (H-C), herb-compound-target (H-C-T), and herb-compound-target-disease (H-C-T-D) networks were established by connecting the corresponding elements [44]. The potential targets of the active compounds contained in GXV and the potential targets of CAD were annotated in Universal Protein (UniProt, https://www.uniprot.org/) [45]. The potential targets shared by the active compounds contained in GXV and CAD were applied for subsequent analysis. All networks were visualized in Cytoscape (version 3.7.1) [46].

### Functional enrichment

Metascape [47] was applied to conduct enrichment analysis (including Gene Ontology (GO) terms [48] and Kyoto Encyclopedia of Genes and Genomes (KEGG) pathways [49]) of targets significantly associated with GXV and CAD, which was different from the previous study [50]. The list of annotations retrieved from the latest version of the Metascape database (last updated on 2019-08-14) was summarized in Table S1. When a term had ≥3 counts, > 1.5 enrichment factors, and *P* < 0.01, it was seen as significant [47, 51]. Moreover, for each given target, protein-protein interaction (PPI) enrichment analysis was carried out with three databases: BioGRID [52], InWeb_InBioMap [53], and OmniPath [54]. MCODE [55] was used to discover closely connected network components in the network containing 3 to 500 components.

### High-performance liquid chromatography (HPLC) fingerprints of GXV

HPLC fingerprints of GXV were performed as described previously [25, 56, 57] with some modifications.

#### Chromatographic conditions

The separations were developed on a Diamonsil-C18 column (4.6 mm × 250 mm, 5 μm) with a constant temperature at 30 °C. The mobile phases consisted of methanol (A) and 0.1% formic acid (B) using a gradient elution as follows: 0 min 5% A, 25 min 20% A, 30 min 40% A, 50 min 60% A, 55 min 95% A, 65 min 95% A, 68 min 5% A, 70 min 5% A, with a 1.0 mL/min flow rate. The injection volume was set as 10 μL, and the detection wavelength was set at 270 nm. All analyses were performed on an Agilent 1290 Infinity HPLC system (Agilent, Santa Clara, USA).

#### Preparation of sample solutions

*Salvianolic Acid B* (111562–201,212, National Institutes for Food and Drug Control), *Salvianolic Acid A* (120,412, Shanghai Ronghe Pharmaceutical Technology Co., Ltd.), *Salvianic Acid A Sodium* (111366–201,305, National Institutes for Food and Drug Control), *Paeoniflorin* (110736–201,136, National Institutes for Food and Drug Control), *Paeonol* (110708–201,407, National Institutes for Food and Drug Control), and *Rosmarinic Acid* (20283–92-5, Nanjing Chunqiu Biological Engineering Co., Ltd.) were accurately weighed and added to methanol at concentrations of 0.1, 0.05, 0.1, 0.1, 0.1, and 0.01 mg/mL as the mixed reference solution.

One-fifth of the prescriptions of GXV (*Codonopsis Radix* (Origin Gansu, Anhui Songshantang Chinese Medicine Co., Ltd.) 40 g, *Ophiopogon japonicus* (Origin Sichuan, Bozhou Yonggang Pieces Factory Co., Ltd.) 20 g, *Schizandra Chinensis Fructus* (Origin Jilin, Bozhou Yonggang Pieces Factory Co., Ltd.) 10 g, *Rehmannia Radix Praeparata* (Origin He’nan, Bozhou Jingwan Traditional Chinese Medicine Pieces Factory) 40 g, *Radix Salviae* (Origin Jiangsu, Anhui Huchuntang Chinese Herbal Pieces Co., Ltd.) 40 g, and *Radix Paeoniae Rubra* (Origin Neimenggu, Bozhou Jingwan Traditional Chinese Medicine Pieces Factory) 40 g,which were authenticated by the chief Chinese pharmacist and met the requirements of the 2015 Chinese Pharmacopoeia) was soaked in a tenfold volume of distilled water at room temperature for half an hour, decocted for 1.5 h, filtered, and then an eightfold volume of distilled water was added into the filter residue for further decocting for an hour. All filtrates were vacuum concentrated as a GXV mixture. The amount of the 1 mL GXV mixture was accurately measured and extracted it with 9 ml of methanol for half an hour. Before HPLC analysis, the volume loss during ultrasound was compensated, and then the extract was filtered by a 0.22 μm membrane filter.

One-fifth of the prescriptions of *Codonopsis Radix* (Origin Gansu, Anhui Songshantang Chinese Medicine Co., Ltd.), *Ophiopogon japonicus* (Origin Sichuan, Bozhou Yonggang Pieces Factory Co., Ltd.), *Schizandra Chinensis Fructus* (Origin Jilin, Bozhou Yonggang Pieces Factory Co., Ltd.), *Rehmannia Radix Praeparata* (Origin He’nan, Bozhou Jingwan Traditional Chinese Medicine Pieces Factory), *Radix Salviae* (Origin Jiangsu, Anhui Huchuntang Chinese Herbal Pieces Co., Ltd.), and *Radix Paeoniae Rubra* (Origin Neimenggu, Bozhou Jingwan Traditional Chinese Medicine Pieces Factory), which were authenticated by the chief Chinese pharmacist and met the requirements of the 2015 Chinese Pharmacopoeia, were accurately weighed, and the preparations were tested according to the preparation method of GXV to obtain the crude drug solution.

#### Method validation

The precision, stability, and repeatability were assessed as described previously [56, 57] and expressed by the relative standard deviation of the average retention time and peak areas. Each sample solution was detected twice in parallel.

## Results

### Investigation of the active phytochemical compounds of GXV

**Fig. 1:**
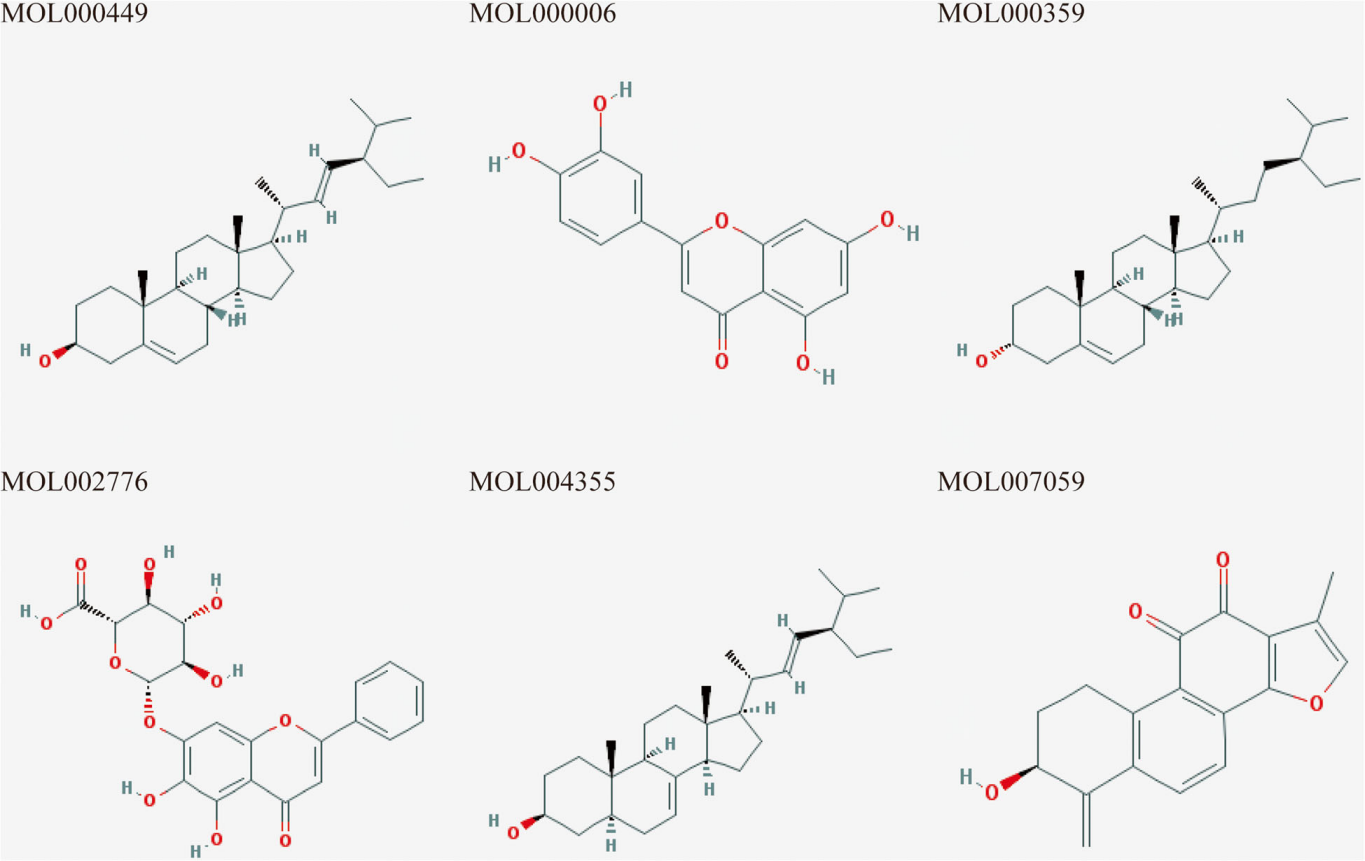
The structure of shared active compounds

**Fig. 2:**
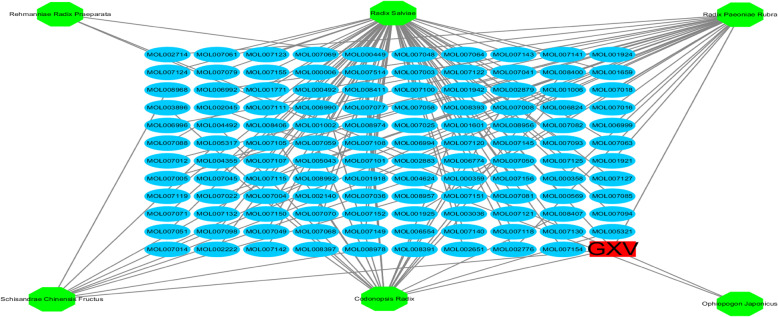
The H-C network of GXV. Red rectangles and green octagons indicate GXV and the six herbal medicines comprising GXV, respectively. Blue ovals indicate the 119 active compounds in GXV

The active phytochemical compounds contained in the six herbs (*Codonopsis Radix*, *Ophiopogon japonicus*, *Schizandra Chinensis Fructus*, *Rehmannia Radix Praeparata*, *Radix Salviae*, and *Radix Paeoniae Rubra*) that comprise GXV were identified from TCMSP, TCMID, and BATMAN-TCM. Consequently, by defining OB and DL, we obtained 21, 1, 8, 2, 65, and 29 compounds for *Codonopsis Radix*, *Ophiopogon japonicus*, *Schizandra Chinensis Fructus*, *Rehmannia Radix Praeparata*, *Radix Salviae*, and *Radix Paeoniae Rubra*, respectively. Some active compounds exist in many kinds of herbs (Fig. 1, Table S2), and 119 active compounds were identified after duplicate removal. To understand the multicomponent pharmacological mechanisms of GXV, we built an H-C network (Fig. 2). The H-C network for GXV was composed of 126 nodes (including GXV, the six herbs, and 119 active compounds) and 132 edges (Fig. 2), indicating that there was much crossover between herbs and compounds.

### Identification of the targets of active phytochemical compounds in GXV

**Fig. 3:**
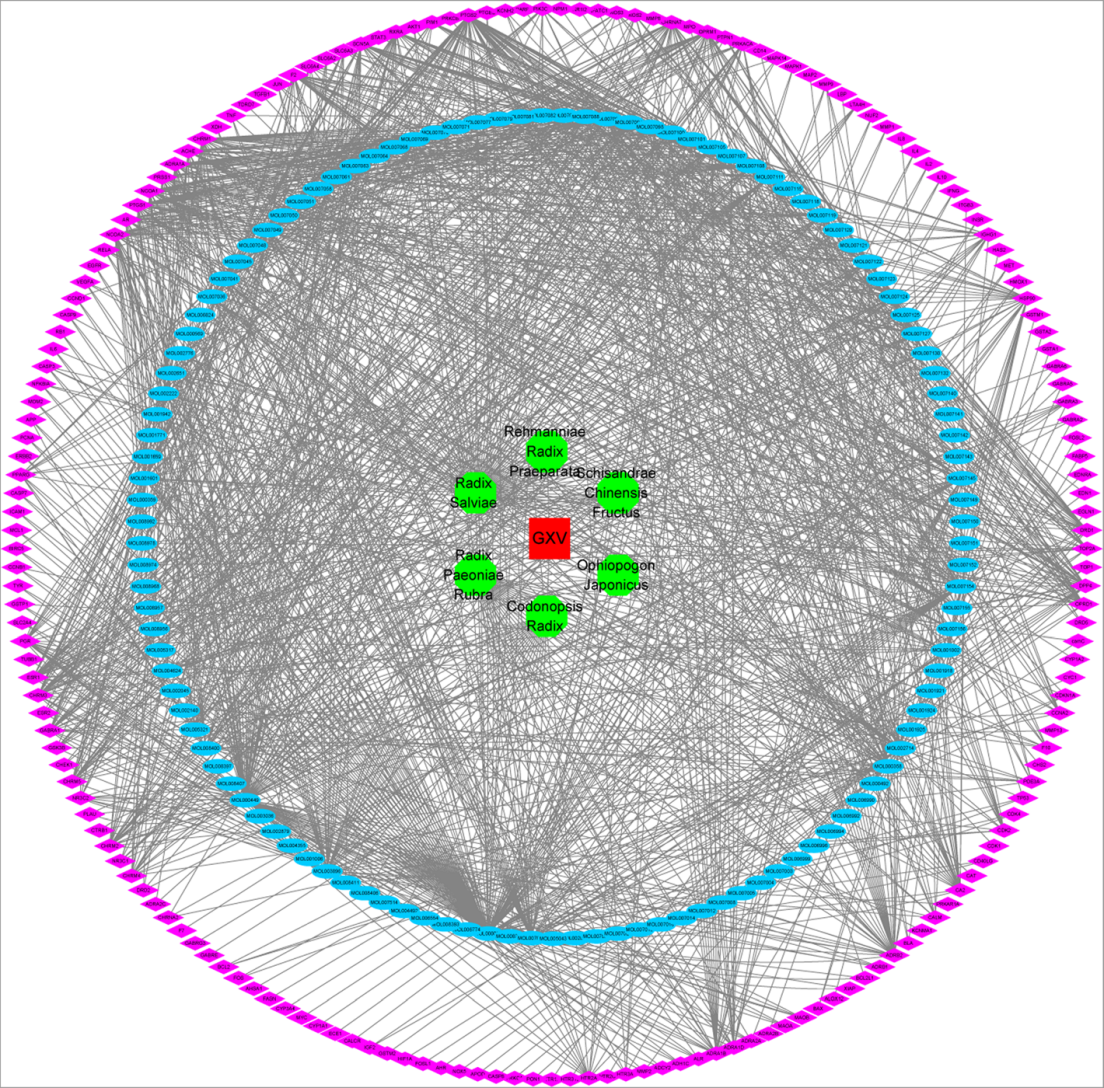
The H-C-T network of GXV. Red rectangles and green octagons indicate GXV and the six herbal medicines comprising GXV, respectively. Blue ovals and purple diamonds indicate the 119 active compounds and 181 targets in GXV, respectively

We obtained 1367 targets for the 119 active phytochemical compounds in GXV (214, 1, 30, 34, 930, and 158 compounds for *Codonopsis Radix*, *Ophiopogon japonicus*, *Schizandra Chinensis Fructus*, *Rehmannia Radix Praeparata*, *Radix Salviae*, and *Radix Paeoniae Rubra*, respectively) by searching the corresponding database. Note that there were 181 targets after duplication removal, suggesting that different active compounds had the same targets. To further understand the multicomponent and multitarget mechanisms of GXV, an H-C-T network was constructed. This network for GXV was composed of 307 nodes (including GXV, the six herbs, 119 active compounds, and 181 targets) and 1499 edges (Fig. 3), investigating the system-level therapeutic properties of GXV.

### Identification of the targets of CAD

To identify CAD-associated human targets, we surveyed diverse databases (GeneCards [37], TTD [38], DrugBank [39], DisGeNET [40, 41], OMIM, TCMSP [30], MalaCards [42], NCBI-Gene, and CTD [43]). Hence, a total of 2336 targets were obtained, with 2028 remaining after deduplication.

### Identification of shared targets of GXV and CAD

After we uploaded 2028 CAD targets and 181 GXV targets, 2026 CAD targets and 181 GXV targets were identified as unique elements, and 2086 unique elements existed in total, which meant that 121 targets were shared by CAD and GXV.

### H-C-T-D network-based analysis of the pharmacological mechanisms of GXV

**Fig. 4:**
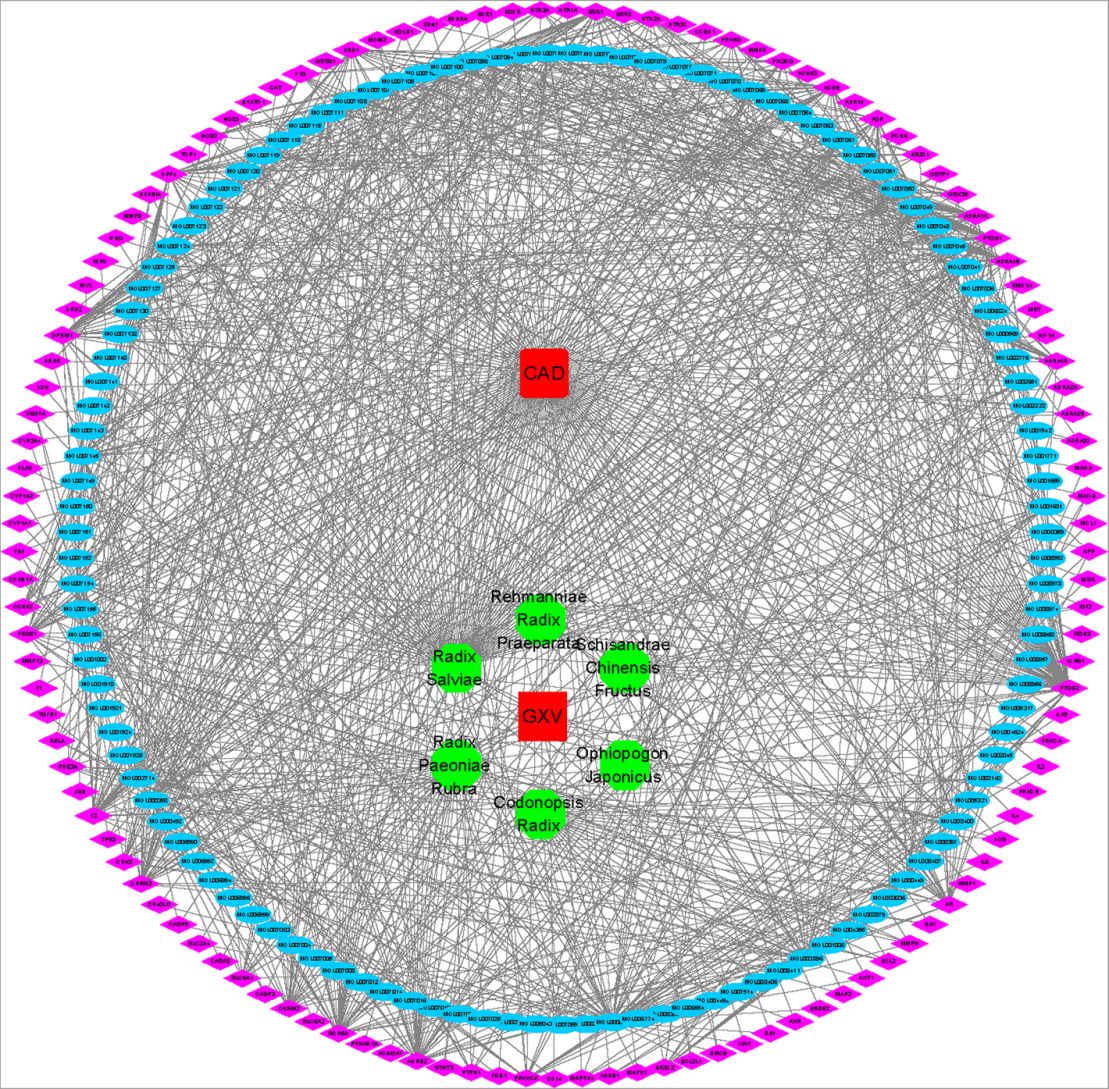
The H-C-T-D network

The potential targets shared by the active compounds contained in GXV and CAD were applied for subsequent analysis. Because there were too many defined CAD targets, we only used the shared targets with GXV to build the H-C-T-D network diagram (Fig. 4). The H-C-T-D network was composed of 248 nodes (including CAD, GXV, the six herbal medicines, 119 active compounds, and 121 shared targets) and 1059 edges.

### Functional enrichment analysis

**Fig. 5:**
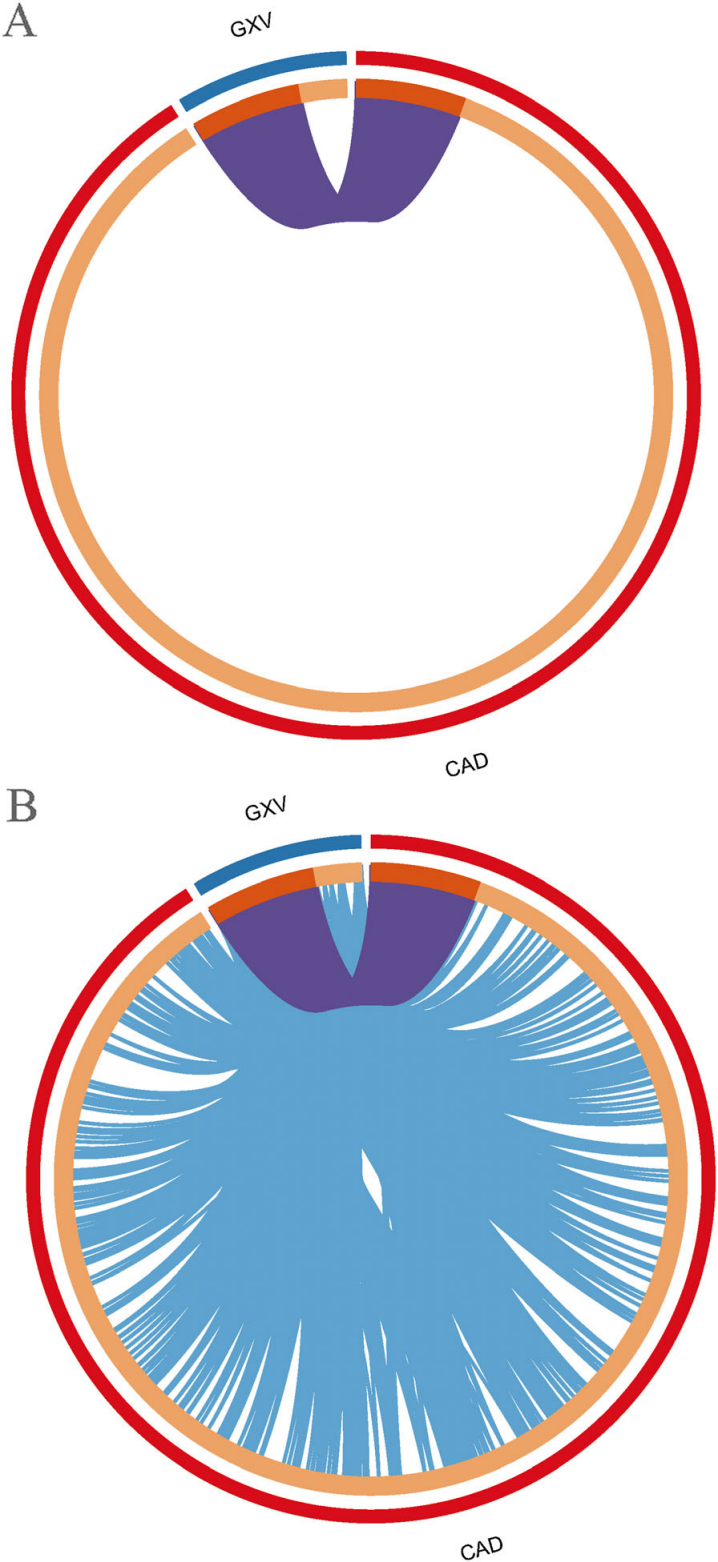
Overlap between GXV and CAD targets. **a** Only at the gene level. On the outside, each arc represents the identity of each gene list. On the inside, each arc represents a gene list, where each gene has a spot on the arc. Dark orange represents the genes that appear in multiple lists and light orange represents genes that are unique to that gene list. Purple lines link the same genes that are shared by multiple gene lists. The greater the number of purple links and the longer the dark orange arcs, the greater overlap among the input gene lists. **b** Including the shared term level. Blue lines link the different genes where they fall into the same ontology term (the term has to be statistically significantly enriched and with a size no larger than 100). Blue links indicate the amount of functional overlap among the input gene lists

**Fig. 6:**
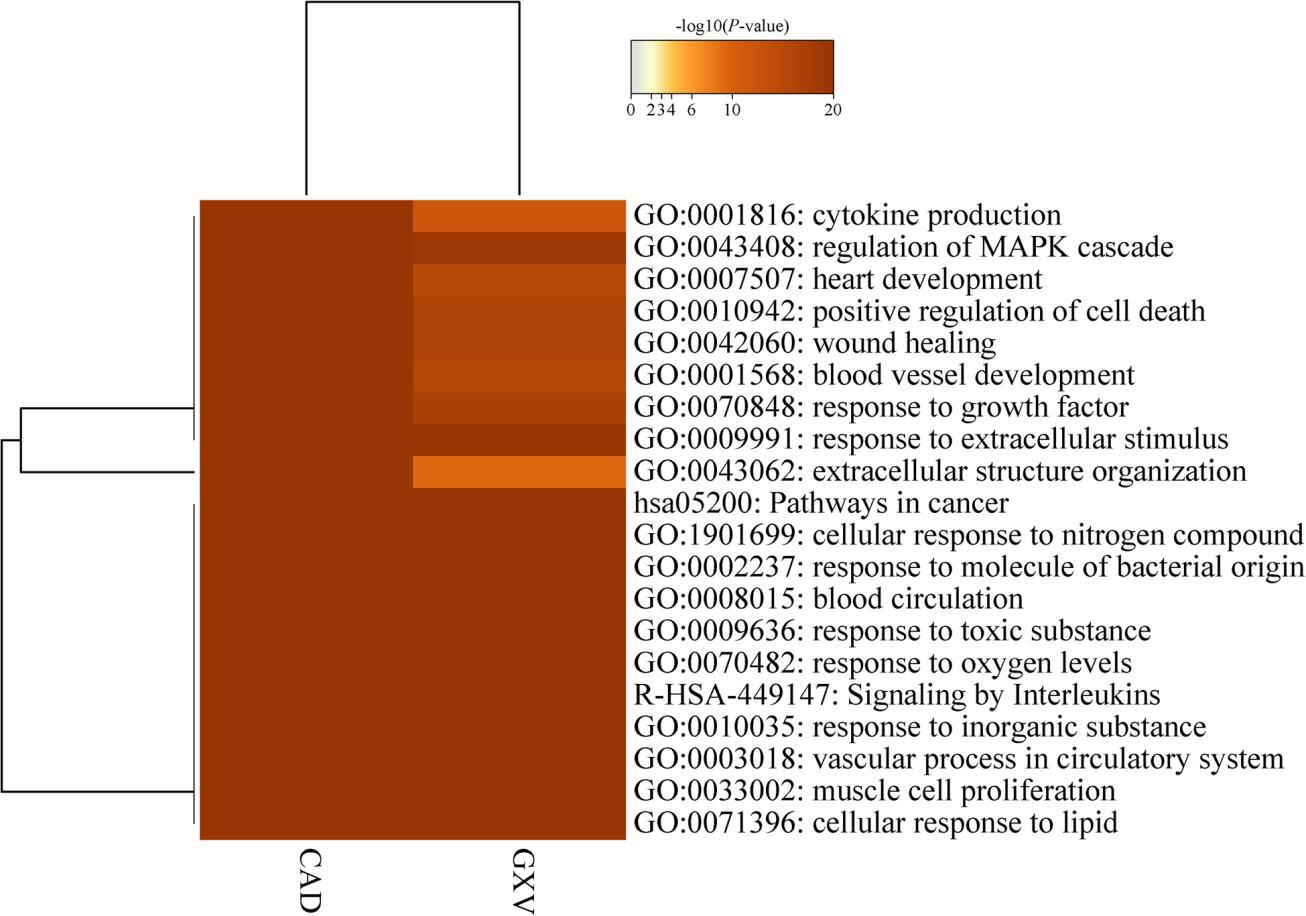
Heatmap of the top 20 enriched terms across targets significantly associated with GXV and CAD, colored by *P*

The targets significantly associated with GXV and CAD that we uploaded were converted into their corresponding gene IDs with the latest version of the database (last updated on 2019-08-14). The overlaps between these targets are shown in a Circos plot [58] (Fig. 5a). Another useful representation is to overlap genes based on their functions or shared pathways. The overlaps between gene lists can be significantly improved by considering overlaps between genes sharing the same enriched ontology terms (Fig. 5b). Only ontology terms that contain less than 100 genes were used to calculate functional overlaps to avoid linking genes using very general annotation. From the heatmap of the top 20 enriched terms across targets significantly associated with GXV and CAD (Fig. 6), we found that the functions for these targets were mainly circulatory system and response, including blood vessel development, blood circulation, cytokine production, heart development, regulation of MAPK cascade, response to growth factor, positive regulation of cell death, signaling by interleukins, cellular response to nitrogen compound, wound healing, response to inorganic substance, cellular response to lipid, response to toxic substance, response to extracellular stimulus, extracellular structure organization, response to molecule of bacterial origin, response to oxygen levels, muscle cell proliferation, and vascular process in circulatory system.

**Fig. 7:**
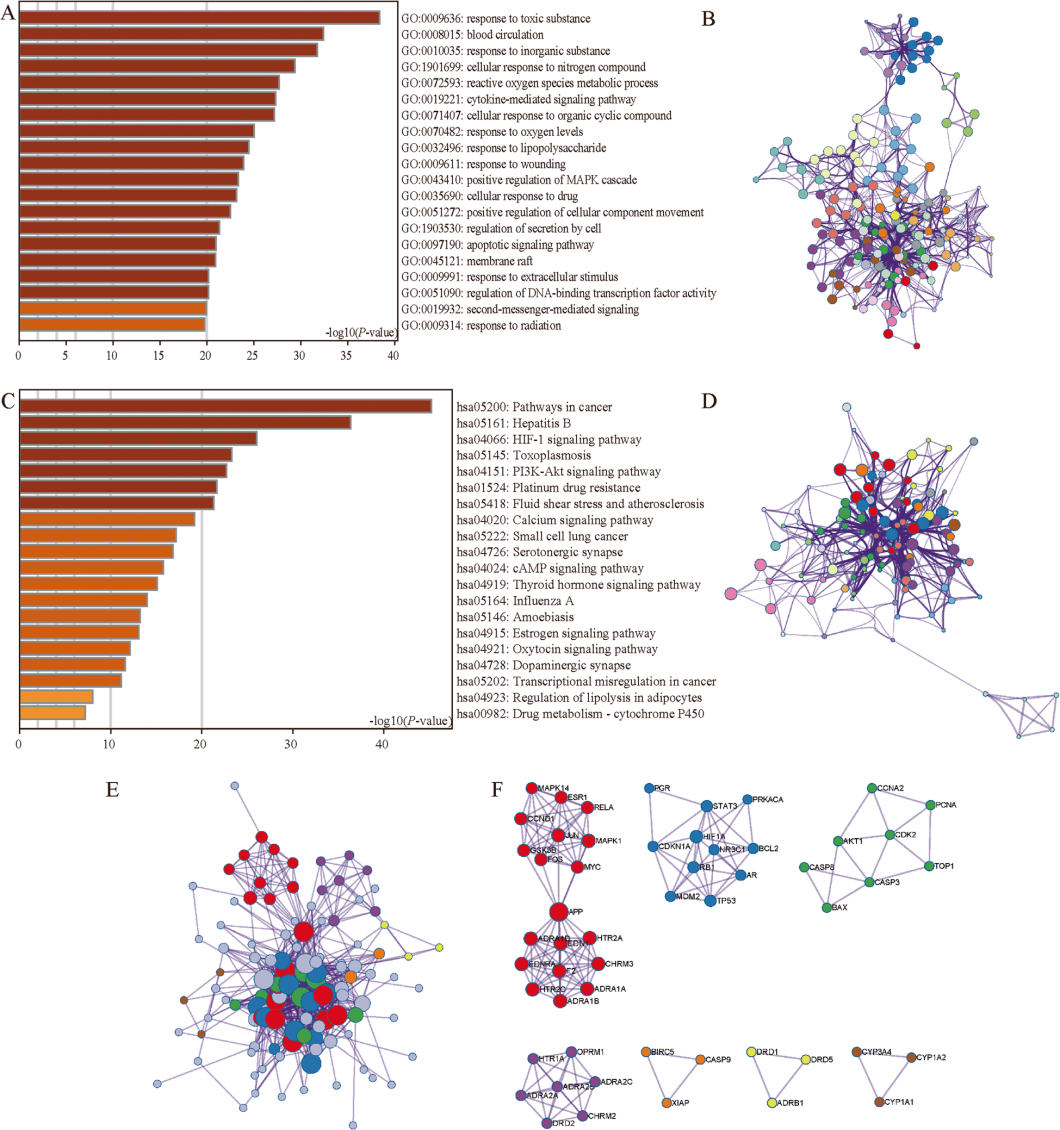
The enrichment analysis of shared targets. **a** Heatmap of GO enriched terms colored by *P*. **b** Network of GO enriched terms colored by cluster, where terms containing different colors tend to have different clusters. **c** Heatmap of KEGG enriched terms colored by *P*. **d** Network of KEGG enriched terms colored by cluster, where terms containing different colors tend to have different clusters. **e** PPI network, where terms containing different colors tend to have different MCODE components. **f** The seven most significant MCODE components form the PPI network

The functions of shared targets were enriched by GO and KEGG from Metascape. The top 20 GO enrichment items were classified into three functional groups: biological process group (19 items), molecular function group (0 items), and cellular component group (1 item) (Fig. 7a). The network of GO enriched terms showed 167 nodes and 1439 edges (Fig. 7b). The shared 121 targets were mainly enriched in response, blood circulation, and apoptosis biological processes such as response to toxic substance, cytokine-mediated signaling pathway, blood circulation, response to inorganic substance, cellular response to nitrogen compound, cellular response to organic cyclic compound, response to wounding, regulation of secretion by cell, positive regulation of MAPK cascade, positive regulation of cellular component movement, apoptotic signaling pathway, response to oxygen levels, reactive oxygen species metabolic process, response to extracellular stimulus, response to lipopolysaccharide, cellular response to drug, regulation of DNA-binding transcription factor activity, second-messenger-mediated signaling, and response to radiation signaling pathway. The cellular components that these genes were involved in were membrane rafts. The top 20 KEGG pathways for the shared targets are shown in Fig. 7c. The network of KEGG enriched terms showed 112 nodes and 1098 edges (Fig. 7d). Among these pathways, the PI3K-Akt signaling pathway, HIF-1 signaling pathway, fluid shear stress and atherosclerosis, calcium signaling pathway, cAMP signaling pathway, serotonergic synapse, thyroid hormone signaling pathway, regulation of lipolysis in adipocytes, and drug metabolism-cytochrome P450 were found to be related to the development of multiple cardiovascular diseases and were involved in CAD development and pathogenesis. These findings support the pharmacological mechanisms of GXV in CAD.

In addition, to better understand the relationship between GXV targets and CAD, we performed a PPI enrichment analysis (Fig. 7e), which indicated 115 nodes and 635 edges. The MCODE networks identified for individual target lists have been gathered and are shown in Fig. 7f. The MCODE results showed that biological function was mainly related to the p53 signaling pathway, neuroactive ligand-receptor interaction, cGMP-PKG signaling pathway, cAMP signaling pathway, apoptosis, calcium signaling pathway, cAMP signaling pathway, neuroactive ligand-receptor interaction, and metabolism of xenobiotics by cytochrome P450.

### Specific chemical identification

The Similarity Evaluation System for Chromatographic Fingerprints of TCMs Software (version 2004A) recommended by the China Food and Drug Administration was used for analysis. Fig. S1A shows the HPLC fingerprints of ten batches of GXV samples (S1–10). Sixteen characteristic common peaks (1–16) were automatically selected in the fingerprints. The relative standard deviation values of the average retention time and peak areas did not exceed 2 and 3%, respectively, indicating that the method is good. The mixed reference solution was used to identify the characteristic common peaks. Six compounds were identified as *Salvianolic Acid B* (14), *Salvianolic Acid A* (15), *Salvianic Acid A Sodium* (5), *Paeoniflorin* (10), *Paeonol* (16), and *Rosmarinic Acid* (12) (Fig. S1B). Through comparison with the crude drug solution, it can be determined that peaks 1, 2, 3, 9, 10, and 16 were derived from *Radix Paeoniae Rubra*; peaks 5, 13, and 14 were derived from *Codonopsis Radix*; peaks 4 and 6 were derived from *Schizandra Chinensis Fructus* (Fig. S1C). The other five peaks (peaks 7, 8, 11, 12, and 15) were unknown, which may be caused by co-fried 6 herbs in the production process.

## Discussion

TCM has a long history in clinical practice [59] and is gradually recognized at home and abroad [60]. CAD is the leading cause of mortality worldwide [61]. Great progress has been made in the treatment of CAD by TCM in recent years [62, 63]. Previous studies have shown that GXV combined with conventional medicine has a better curative effect than conventional medicine alone in CAD [8, 9]. GXV can significantly improve the clinical symptoms [11, 12], reduce the incidence of angina and the amount of nitroglycerin [11], improve the 24-h Holter myocardial ischemia load and heart rate variability [12], ejection fraction [10], stroke volume and cardiac output based on echocardiography [13]. Additionally, GXV reduces total cholesterol, triglycerides, and low density lipoprotein cholesterol [11], and increases high-density lipoprotein cholesterol [11] and six-minute walking distance [13]. The underlying mechanisms are also being explored. GXV lowers the serum levels of NT-pro BNP, hs-CRP, MMP-9, Ang II and ET-1 in patients with CAD [10].

In vivo experiments showed that GXV increases the ejection fraction and fractional shortening and reduces the left ventricular mass index [14, 15, 18] and reduces the levels of IL-6, TNF-α and other inflammatory factors [14, 15] in rats with acute myocardial infarction by inhibiting the NF-κB pathway [18, 19].

Virtual screening and network pharmacology are effective ways to find the relationship between multiple components and targets of TCM [64, 65]. In the present study, we identified the systemic mechanisms of GXV in the treatment of CAD by these approaches, which provides a new strategy to study the potential active components and targets of TCM [66, 67]. Our main findings can be summarized as follows: (I) 119 potentially active compounds from GXV had an interaction with 121 CAD-related targets, showing therapeutic activity; (II) functional enrichment analysis revealed that the targets from GXV were involved in various CAD-associated biological processes, such as cytokine-mediated signaling pathway, blood circulation, cellular response to nitrogen compound, response to wounding, regulation of secretion by cell, positive regulation of MAPK cascade, positive regulation of cellular component movement, apoptotic signaling pathway, response to oxygen levels, reactive oxygen species metabolic process, response to extracellular stimulus, response to lipopolysaccharide, cellular response to drug, and regulation of DNA-binding transcription factor activity; (III) the CAD-associated targets of GXV were significantly enriched in diverse pathways, including the PI3K-Akt signaling pathway, HIF-1 signaling pathway, fluid shear stress and atherosclerosis, calcium signaling pathway, cAMP signaling pathway, serotonergic synapse, thyroid hormone signaling pathway, regulation of lipolysis in adipocytes, and drug metabolism-cytochrome P450, which are associated with CAD.

GXV is composed of six herbal medicines containing 119 active compounds that interact with 121 CAD-related targets. These herbs and chemical constituents of GXV have been reported to be beneficial to CAD. *Codonopsis Radix* shares immunomodulation effects [68, 69]. Radix Codonopsis polysaccharide, an active compound in *Codonopsis Radix*, could maintain the T-cell balance against hydrocortisone disturbance [70]. Choushenpilosulynes A-C, isolated from *Codonopsis Radix*, can inhibit the expression of SQLE involved in lipid metabolism [71]. ShenMai injection, prepared from *Panax ginseng* and *Ophiopogon japonicus*, is used as an add-on therapy for CAD [72, 73]. The main components of *Ophiopogon japonicus* exhibit various pharmacological activities, such as cardiovascular protection [74–77], anti-inflammation [74, 76, 78–80], anti-oxidation [74–76, 81], mitochondrial function preservation [81], apoptosis inhibition [75, 81], and immunomodulation [74, 77]. The extract of *Ophiopogon japonicus* decreased ICAM-1 and VCAM-1 to play an endothelial protective role from oxidative damage and dysfunction [82]. In addition, it also inhibited proliferation [82]. *Ophiopogon japonicus* has a regulatory impact on the cAMP signaling pathway, WNT signaling pathway, and PI3K-AKT signaling pathway by targeting HSPA8, TP53, and VEGFA [83]. The key cardioprotective mechanisms of *Schizandra Chinensis Fructus* and its active ingredients have been demonstrated to include anti-oxidation [84–87], suppression of apoptosis [84, 88], and anti-inflammation [84, 86, 87]. *Schizandra Chinensis Fructus* increases antioxidant capacity and improves endothelial dysfunction to ameliorate the extent of atherosclerosis [85]. *Schizandra Chinensis Fructus* extracts induce apoptosis via the ROS-mediated/mitochondria-dependent pathway and JNK/p38 MAPK activation [89]. In addition, *Schizandra Chinensis Fructus* was found to facilitate PI3K-AKT activation and inhibit the expression of NOX2 in AMI mice and oxygen-glucose deprivation-treated H9c2 cells [90]. *Rehmannia Radix Praeparata* had efficient detectable antioxidant activity [91], and the PI3K-Akt and MAPK signaling pathways were found in the pathway analysis for CAD on *Rehmannia Radix Praeparata* [92]. In addition, extraction from *Rehmannia Radix Praeparata* regulated the IGF-1/PI3K/mTOR signaling pathways [93]. The compounds from *Radix Salviae* showed various pharmacological activities, such as anti-inflammation [94–96], antioxidation [94, 96, 97], direct thrombin inhibitory effects with a dose-effect relationship [98], antiproliferation [99], improvement in microcirculatory disturbances [96], blocking of calcium inflow and prevention of calcium overload [96], and anti-atherogenesis [94], and its mechanisms may be related to activating the Nrf2 pathway [97] and NF-κB modulation [95]. *Radix Paeoniae Rubra* has antiinflammatory [100], antiproliferation [101], antiapoptosis [102], immunoregulatory [100], scavenging free radicals [103], regulation of lipid metabolism [104], antifibrosis [103], and myocardial protection [102] properties via the NF-κB [100], MAPK [100], PI3K/Akt/mTOR [102], and TGF-β/Smad [103] signaling pathways. Moreover, *Radix Paeoniae Rubra* extract had an inhibitory effect on thrombus formation, and the antithrombotic effects were associated with the regulation of vascular endothelium active substances, activating blood flow and anticoagulation effects [105].

Fingerprints can comprehensively reflect the types and quantities of chemicals contained in medicines, thereby effectively evaluating and controlling the quality of TCM [106]. In this study, HPLC was used to establish the fingerprints of GXV, which can reflect the quality of the overall characteristics and provide a basis for overall quality control, thereby improving the stability and ensuring the safety and effectiveness of clinical medication. It also laid the foundation for basic research on the medicinal substances of GXV.

In conclusion, these previous findings support the potential role of herbal and chemical constituents of GXV in the treatment of CAD. Furthermore, we have validated the new potential therapeutic targets and underlying molecular mechanisms of GXV against CAD, which might provide a reference for its future application in cardiovascular diseases [29, 107, 108]. More studies are needed to further validate the therapeutic properties of GXV.

The results presented in this study improved our understanding of GXV, which is prescribed for CAD. The system mechanisms of GXV for CAD were identified through 119 major active ingredients and 121 candidate targets. In particular, those candidate targets were highly correlated with CAD in our functional enrichment results. These studies indicate the feasibility of the predicted biological processes and pathways. However, the regulation of GXV on key biological processes and key pathways in CAD needs further basic and clinical research confirmation. The findings of potential key targets may provide new clues for CAD treatments with GXV.

## Conclusions

Via the method of integrative virtual screening and network pharmacology, our study predicts the targets of the ingredients of GXV and explores the underlying mechanisms of the potential anti-CAD effects, providing a complementary and alternative therapy for CAD. We have reasons to believe that the potential mechanisms are direct or indirect synergy of multitarget and multipathway efforts. However, more experimental validation is essential to reveal the effect of GXV against CAD.

## Supplementary Information

**Additional file 1 Table S1**. Gene annotations extracted.

**Additional file 2 Table S2**. Details of compounds in various herbs.

**Additional file 3 Fig. S1**. The HPLC fingerprints of GXV. A) The fingerprints of 10 batches of GXV. B) The fingerprints of GXV (above) and mixed reference solution (below). C) The fingerprints of crude drugs.

## Data Availability

All data generated or analysed during this study are included in this published article and its supplementary information files.

## Abbreviations

CAD: Coronary artery disease
DL: Drug likeness
GO: Gene Ontology
GXV: Guanxin V
H-C: herb-compound
H-C-T: Herb-compound-target
H-C-T-D: Herb-compound-target-disease
HPLC: High performance liquid chromatography
KEGG: Kyoto Encyclopedia of Genes and Genomes
OB: Oral bioavailability
PPI: Protein-protein interaction
TCM: Traditional Chinese medicine
UniProt: Universal Protein
