# Supplementary material for: Virtual screening and network pharmacology-based synergistic mechanism identification of multiple components contained in Guanxin V against coronary artery disease
Source: BMC Complement Med Ther. 2020 Nov 13;20:345. doi: 10.1186/s12906-020-03133-w (PMC7664106; doi:10.1186/s12906-020-03133-w)
Supplement: Supplementary file 1 — Additional file 1 Table S1. Gene annotations extracted. [file 12906_2020_3133_MOESM1_ESM.docx]

Table S1. Gene annotations extracted.

| Name | Type | Description |
| --- | --- | --- |
| Gene Symbol | Description | Primary HUGO gene symbol. |
| Description | Description | Short description. |
| Biological Process (GO) | Function/Location | Descriptions summarized based on gene ontology database, where up to three most informative GO terms are kept. |
| Protein Function (Protein Atlas) | Function/Location | Protein Function (Protein Atlas) |
| Subcellular Location (Protein Atlas) | Function/Location | Subcellular Location (Protein Atlas) |
