# Supplementary material for: Virtual screening and network pharmacology-based synergistic mechanism identification of multiple components contained in Guanxin V against coronary artery disease
Source: BMC Complement Med Ther. 2020 Nov 13;20:345. doi: 10.1186/s12906-020-03133-w (PMC7664106; doi:10.1186/s12906-020-03133-w)
Supplement: Supplementary file 2 — Additional file 2 Table S2. Details of compounds in various herbs. [file 12906_2020_3133_MOESM2_ESM.docx]

Table S2. Details of compounds in various herbs.

| Molecule ID | Molecule name | Pubchem Cid | Herbs |
| --- | --- | --- | --- |
| MOL000449 | Stigmasterol | 5280794 | Codonopsis Radix, Rehmannia Radix Praeparata, and Radix Paeoniae Rubra |
| MOL000006 | Luteolin | 5280445 | Codonopsis Radix, Radix Salviae |
| MOL000359 | Sitosterol | 12303645 | Rehmannia Radix Praeparata, and Radix Paeoniae Rubra |
| MOL002776 | Baicalin | 64982 | Radix Salviae, and Radix Paeoniae Rubra |
| MOL004355 | Spinasterol | 5281331 | Codonopsis Radix, and Radix Paeoniae Rubra |
| MOL007059 | 3-beta-Hydroxymethyllenetanshiquinone | 5318290 | Codonopsis Radix, Radix Salviae |
