# Supplementary figures and images for: Virtual screening and network pharmacology-based synergistic mechanism identification of multiple components contained in Guanxin V against coronary artery disease
Source: BMC Complement Med Ther. 2020 Nov 13;20:345. doi: 10.1186/s12906-020-03133-w (PMC7664106; doi:10.1186/s12906-020-03133-w)

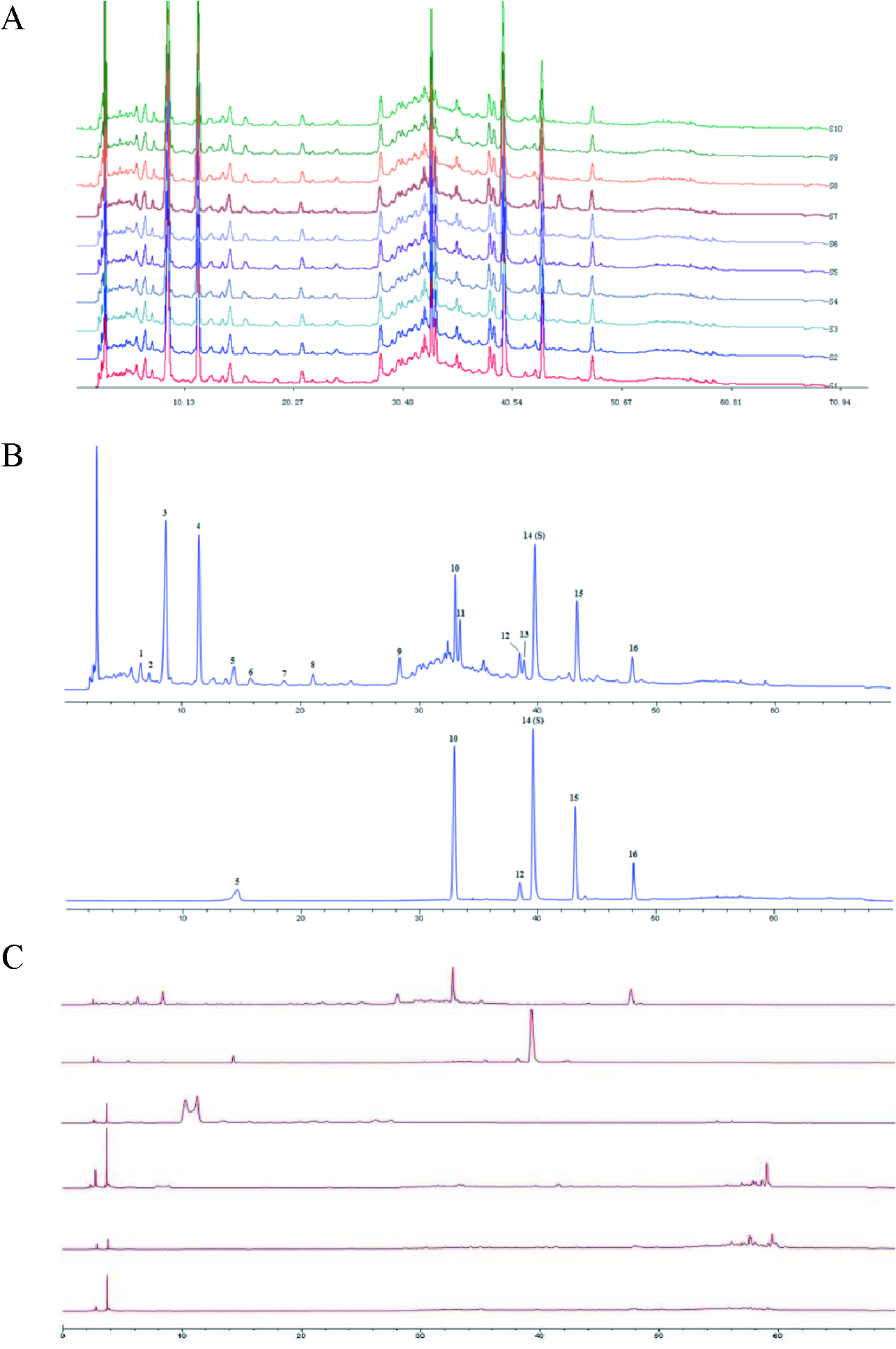

Supplement: Supplementary file 3 — Additional file 3 Fig. S1. The HPLC fingerprints of GXV. A) The fingerprints of 10 batches of GXV. B) The fingerprints of GXV (above) and mixed reference solution (below). C) The fingerprints of crude drugs. [file 12906_2020_3133_MOESM3_ESM.tif]
